# Prediction of Lactational Milk Yield of Cows Based on Data Recorded by AMS during the Periparturient Period

**DOI:** 10.3390/ani11020383

**Published:** 2021-02-03

**Authors:** Piotr Kliś, Dariusz Piwczyński, Anna Sawa, Beata Sitkowska

**Affiliations:** 1 Lely Center Bydgoszcz, Lisi Ogon, 86-065 Łochowo, Poland; pklis@byd.lelycenter.com; 2 Department of Animal Biotechnology and Genetics, Faculty of Animal Breeding and Biology, UTP University of Science and Technology, 85-084 Bydgoszcz, Poland; beatas@utp.edu.pl; 3 Department of Animal Breeding, Faculty of Animal Breeding and Biology, UTP University of Science and Technology, 85-084 Bydgoszcz, Poland; sawa@utp.edu.pl

**Keywords:** milk performance, dairy cattle, correlation, data mining, forecasting

## Abstract

**Simple Summary:**

Barns equipped with the automatic milking system (AMS) record huge amounts of data on milk flow rate, milk yield and composition, milk temperature, amount of concentrate intake and rumination time. Our study attempted to use this information, recorded during the periparturient period (divided into subperiods: second (14–8 days) and first (7–1 days) week before calving; 1–4, 5–7, 8–14, 15–21 and 22–28 days of lactation), to predict lactation milk yield in Polish Holstein–Friesian cows. In the first stage of statistical analysis, coefficients of simple correlation between lactation milk yield and AMS parameters were calculated. We found that prediction of lactation milk yield based on individual pieces of data may be ineffective—the calculated coefficients of correlation were low or moderate. In the next step of data analysis, we used a modern data mining technique in the form of decision trees. Based on the graphic, easy-to-interpret decision tree, we concluded that the highest lactation yield is to be expected for cows with completed lactations (survived until the next lactation), which were milked 4.07 times per day on average in the 4th week of lactation.

**Abstract:**

Early prediction of lactation milk yield enables more efficient herd management. Therefore, this study attempted to predict lactation milk yield (LMY) in 524 Polish Holstein–Friesian cows, based on information recorded by the automatic milking system (AMS) in the periparturient period. The cows calved in 2016 and/or 2017 and were used in 3 herds equipped with milking robots. In the first stage of data analysis, calculations were made of the coefficients of simple correlation between rumination time (expressed as mean time per cow during the periparturient period: second (14–8 days) and first (7–1 days) week before calving, 1–4, 5–7, 8–14, 15–21 and 22–28 days of lactation), electrical conductivity and temperature of milk (expressed as means per cow on days 1–4, 5–7, 8–14, 15–21 and 22–28), amount of concentrate intake, number of milkings/day, milking time/visit, milk speed and lactation milk yield. In the next step of the statistical analysis, a decision tree technique was employed to determine factors responsible for LMY. The study showed that the correlation coefficients between LMY and AMS traits recorded during the periparturient period were low or moderate, ranging from 0.002 to 0.312. Prediction of LMY from the constructed decision tree model was found to be possible. The employed Classification and Regression Trees (CART) algorithm demonstrated that the highest lactation yield is to be expected for cows with completed lactations (survived until the next lactation), which were milked 4.07 times per day on average in the 4th week of lactation. We proved that the application of the decision tree method could allow breeders to select, already in the postparturient period, appropriate levels of AMS milking variables, which will ensure high milk yield per lactation.

## 1. Introduction

From the economic point of view, milk yield is the most important productive trait of cows. It increases consistently due to effective breeding work and improved welfare. At the same time, increases in cow herd size [1], changes in herd management procedures and replacement of the time-consuming and labor-intensive conventional milking system (CMS) with the automatic milking system (AMS) have been observed in many countries. Milking robots are constantly being improved and equipped with additional functionality. Unlike the CMS, the AMS not only records numerous data (milking parameters and milk characteristics—milk composition and cytological quality, electrical conductivity, temperature) during successive visits of the cows to the milking robot [2], but also allows for easier and more thorough monitoring of daily rhythms and behaviors of the cows during the entire production cycle [3].

The AMS also monitors rumination [4], which is essential to the normal digestive function of cattle. Rumination time provides extensive information about the quality of feed offered, but can also be used to predict the cow’s milk yield [5]. Higher yielding cows require more feed, which Stone et al. [6] believe can increase rumination time compared to lower yielding cows. Positive correlations between rumination time and milk yield of early lactation cows were reported by Antanaitis et al. [4], Soriani et al. [5], Calamari et al. [7], Liboreiro et al. [8], but the authors did not analyze the incidence of these correlations in particular weeks after calving. Box time, milking time and milking speed are important for utilizing AMS efficiently, because short milking time and the ability to quickly leave the AMS after the last teat cup is removed are desirable traits [9].

Modern statistical data analysis techniques are needed to process the enormous amount of data recorded by the AMS. The results of many studies provide evidence that the decision tree technique, which is one of the data mining approaches, is a useful method to explore data in this respect.

Decision tree techniques have found application in dairy cow breeding to study mastitis [10], predict milk yield [11], parturition process [12] and reproduction in cows [13]. The advantages of decision trees (composed of the root, trunk, branches and leaves) [14] are that they are intuitive and it is easy to interpret the data shown as simple graphical models for analyzing the effect of single factors in the model but also their interactions. The obtained results could be an excellent tool for managers of AMS herds, allowing them to predict events and take decisions for improved performance of the cows. Such an approach may help to reveal factors that had previously been disregarded when predicting lactational milk yield. Considering the complex nature of the problem and the search for new solutions to predict milk yield as soon as possible after calving, the present study accounts for data from the periparturient period (two weeks before and four weeks after calving), which is considered critical for the cow’s production cycle [15]. Two weeks before parturition, the amount of feed intake decreases. In turn, the first weeks postcalving are crucial for whole-lactation efficiency, because milk production increases from zero to the maximum level.

For breeding practice, it is important to predict lactation milk yield as early as possible; our research hypothesis was that there are relationships between some AMS data about cows in the perinatal period, which reflect, e.g., digestive system function (chewing time), mammary gland health (electrical conductivity and temperature of milk) and other factors (amount of concentrated feed consumed in AMS, number of milkings/day, milking time/visit) and their lactation efficiency.

The aim of the study was to determine the possibility of using AMS data for periparturient cows to predict their lactation milk yield.

## 2. Materials and Methods

The study material was obtained from Lely T4C data and herd management system. The analysis covered data for 18,055 milkings of 524 Polish Holstein–Friesian (PHF) cows, which calved in 2016 and/or 2017 and were used in 3 herds equipped with the Lely Astronaut A4 automatic milking system (AMS) (Lely Industries N.V.: Cornelis van der Lelylaan 1, Maassluis, The Netherlands) (Table 1). Milk yield for the study cows was 12,103 kg per lactation (13,212 kg for cows survived until the next lactation and 9019 kg for cows culled during lactation).

The following 24-h data were collected for each cow:

1. Data on rumination time per day (min) during the periparturient period (14 days before and 28 days after calving)
2. Data on milking parameters per day during 1–28 days of lactation:
  (a) quantity of programmed concentrate feed (kg/day)
  (b) amount of concentrate intake (kg/day)
  (c) number of milkings/day
  (d) number of refusal milkings/day
  (e) AMS box time (s/visit) = total time spent by cow in the AMS box per day/number of visits per day
  (f) milking time (s/visit) = total milking time per day/number of visits
  (g) time of colostrum/milk flow from the udder quarter (s/visit) = total time of colostrum/milk flow per day from individual quarters/(number of quarters × number of milkings per day)
  (h) dead milking time for udder quarters (s/visit) = total dead milking time per day in different quarters/number of quarters × number of milkings per day
3. Data on colostrum/milk traits per day during 1–27 days of lactation
  (a) electrical conductivity of colostrum/milk (μS/cm) = total electrical conductivity from
  (b) colostrum/milk temperature (°C) = total colostrum/milk temperatures in all milkings per day/number of milkings
  (c) colostrum/milking speed (kg/min) = total colostrum/milking speed in all milkings per day/number of milkings
  (d) yield of colostrum/milk per day (kg) = total from all milkings
  (e) fat content (%) = mean from 24-h visits
  (f) protein content (%) = mean from 24-h visits
  (g) different quarters per day/number of quarters × number of milkings

The analysis of the collected numerical data started by calculating coefficients of simple correlation between rumination time (expressed as mean time per cow during the periparturient period: second (14–8 days) and first (7–1 days) week before calving, 1–4, 5–7, 8–14, 15–21 and 22–28 days of lactation), electrical conductivity and temperature of milk (expressed as means per cow on days 1–4, 5–7, 8–14, 15–21 and 22–28), amount of concentrate intake in the AMS, number of milkings/day, milking time/visit, milk speed and lactation milk yield. For this purpose, the CORR procedure was used [16].

In the next step of the statistical analysis, the decision tree technique was employed to determine factors responsible for lactation milk yield. The decision tree modelling started by splitting the data set (524 cows) into a training (60%) and validation set (40%). Cows were assigned to the training and validation sets by the random sampling method. The training set contained data serving to detect possible relationships between variables. It was used for preliminary estimation of the model’s parameters. In turn, the validation set served to adjust the model’s parameters, which were estimated based on the training set, and its use improved the model’s predictive capability. When constructing the decision tree, the minimal final node size was set to 30 and the maximum depth size to 5. This approach was aimed to avoid overfitting the tree to training data, which could lead to random correlations in the validation set.

**Figure 1:**
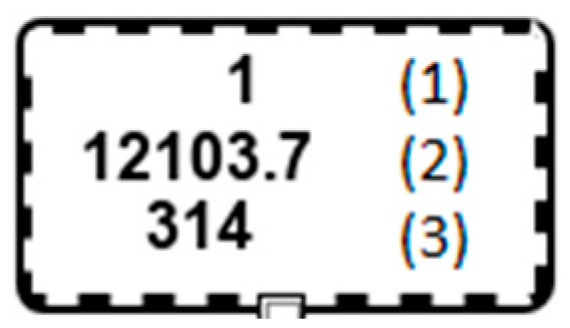
Description of node for kg of milk per lactation.

The CART (Classification and Regression Trees) algorithm, employed to construct the decision tree, used variance reduction as a criterion for division of the data set (SAS Institute Inc., 2014]). When constructing the tree, we accounted for all the data recorded by AMS during the periparturient period (Table 2 and Table 3), with a division into predefined time intervals (14–8, 7–1 days before calving; 1–4, 5–7, 8–14, 15–21 and 22–28 days of lactation) and additional variables: herd, year and calving season. Each node or leaf in the decision tree contained the following information: node ID (1), mean lactation milk yield (12,103.7 kg milk) (2) and number of observations in node or leaf (314) (3) (Figure 1).

The ranking of variables in terms of their importance in creating data set splits was prepared based on the “importance” measure (SAS Institute Inc., Cary, NC, USA, 2014). A statistical analysis was conducted using the Enterprise Miner 15.1 software included in the SAS package [16].

## 3. Results and Discussion

During the colostral period, rumination time of the cows averaged 327 min/day (Table 2). The relatively short rumination time during this period was likely the effect of parturition and the associated parturition stress [17] or social stress due to change in herd hierarchy after the cows were moved from the dry-off group to the lactating group [18]. The amount of programmed concentrate feed was 3.26 kg, and the amount of feed ingested was 2.56 kg. There were 1.82 successful milkings and 1.15 refusal milkings. Visit time per cow per AMS box time was 419 s, with milking time of 322 s/visit. Quarter milking time was 222 s/visit. The difference between milking time and quarter milking time could be explained by the fact that milking yield and milking time are different for each quarter. In addition, udder milking time covers the time from the start to the end of colostrum flow, and colostrum does not start and end to flow from the quarters at the same time. The “blind” milking time was 13.48 s/visit. Hovinen and Pyörälä [19] in the review paper showed that teat preparation of AMS was sufficient for milk ejection, independent of the teat preparation method, that is, brushing or cleaning with a cup of warm or cold water.

The secretion rate of colostrum averaged 2.78 kg/min. Daily yield of colostrum was around 18 kg and it had a high content of basic nutrients: 4.85% protein and 4.89% fat. Healthy udders were reflected in the electrical conductivity of colostrum, which was less than 70 μS/cm. According to Ontsouk et al. [20], increased the electrical conductivity values of milk immediately after calving are generally due to increased somatic cell count, especially during the colostral subperiod, which mainly results from udder morphological structure, which is characterized by high sensitivity and permeability of tissue. Colostrum temperature averaged 39 °C, which is similar to the value of 38.7 ± 1.1 °C reported by King et al. [21].

During 5–28 days of lactation, rumination time was around 445 min/day (Table 3). This value falls within the typical range reported in the literature (340–540 min/day) [4,5,21,22] and shows that the cows were in a good health condition. Rumination time increased in relation to the colostral period, which is consistent with the findings of other authors [21]. During early lactation, cows generally show increased appetite, resulting in higher feed intake. In our study, the amount of programmed concentrate feed offered in the AMS and ingested by the cows was 6.19 and 5.56 kg/day, respectively. The stay of the cows in the box was 446 s/visit. In the study by Sitkowska et al. [23], the time spent in the milking robot was 361 s for primiparous cows and 383 s for multiparous cows. Compared to the colostral period, there were increases in the number of milkings/day (up to 2.78) and in the number of refusals milkings (up to 1.70). The observed number of milkings per day is in agreement with the range (2.5–2.9) reported in the literature for AMS-milked cows [24,25,26,27,28]. Cows released milk after an average of 14 s, milking time was 352 s/visit, and milk flow time from the udder was 250 s/visit. In the study by Sitkowska et al. [23], milking time/visit during the first 100 days of lactation was 268 s, whereas Edwards et al. [28] reported that milking during the first 60 days of lactation took 416 s/visit. Compared to the colostral period, milking speed slightly increased (up to 2.81 kg/min), milk electrical conductivity decreased (to 68.77 μS/cm), and milk temperature, like in the study of King et al. [21], remained similar at 39.03 °C. Similar values (2–2.5 kg/min) for milking speed were reported by Gäde et al. [29] and Bogucki et al. [25], and higher values (3–4 kg/min) were observed by Carlström et al. [30], who concluded that milking time and milk flow rate determine the cow’s milkability. During 5–28 days of lactation, cows yielded over 35 kg milk/day, which is more than daily yield of AMS-milked cows in the EU countries and the USA in the years 2014–2017 reported by Piwczyński et al. [11], who showed the highest value in the US population (33.5 kg/day) and the lowest value in Lithuania (22.7 kg/day). Milk fat content averaged 4.00% and average protein content was 3.54%. It is assumed that in PHF cows, normal milk protein content ranges from 3.2 to 3.6%, and that of fat from 3.5 to 4.5%. The fat/protein ratio was 1.14. This value is considered normal based on the fat/protein ratio of 1.1–1.4 reported by Guliński and Kłopotowska [31] as being indicative of proper feeding.

Table 4 presents the coefficients of linear correlation between lactation yield and AMS-recorded periparturient traits. A weak but statistically significant correlation was found between rumination time in the periparturient period and lactation milk yield. Lengthening rumination time, both during the dry-off period and in the first weeks of lactation, had a positive effect on milk yield. The magnitude of these relationships depended on the week of the periparturient period—it was the weakest during the colostral period (r = 0.097^x^) but increased to over 0.3 in the subsequent weeks. These results are consistent with the study by Antanaitisi et al. [4], in which rumination time was positively correlated with milk yield (r = 0.384, *p* < 0.001). Other authors [5,7,8] also noted a positive correlation between rumination time in early lactation cows and milk yield, but the cited studies failed to account for the effect of week of lactation.

The correlation coefficients between AMS-recorded periparturient parameters of udder health (electrical conductivity and milk temperature) and lactation milk yield, regardless of the week, had low, negative values (most often <−0.1), sporadically statistically significant (Table 4). Week of lactation caused small differences in the coefficients of correlation between electrical conductivity and lactation yield. During successive weeks of lactation, the magnitude of these correlations increased (r = −0.060 vs. r = −0.086^xx^), which suggests milk production losses. Higher values (r = −0.32^xx^) of the correlation coefficients between electrical conductivity and daily milk yield were obtained by Boas et al. [32]. In turn, Neamț et al. [33] showed no statistically significant effect of milk electrical conductivity on milk production.

Further information provided by AMS milking and used in the study to determine its usefulness for predicting the milk yield included the amount of concentrate intake in AMS, the number of milkings/day, milking time/visit, milking speed. The coefficients of correlation between the amount of concentrate feed intake/day in the AMS and lactation milk yield were positive and significant, and the magnitude of their relationship increased over subsequent weeks of lactation (0.109^xx^ vs. 0.197^xx^) (Table 4). The relationships between the number of milkings/day and lactation milk yield were positive and significant—their magnitude was highest in the second week of lactation (r = 0.301^xx^) and ranged from 0.252 to 0.280 in the other periods. Due to the stimulation of milk production associated with frequent milking, and the individual rewarding with feed in the AMS, it is possible to milk out the cows from the start of lactation. When using free movement of the cows preferred by Lely, the cow itself decides the time of milking or resting. Our study showed that milking time/visit has a positive effect (*p* ≤ 0.05 and *p* ≤ 0.01) on lactation milk yield, and the magnitude of the relationships increased over the subsequent weeks of lactation (0.116 vs. 0.174 and 0.062 vs. 0.134). In New Zealand studies, genetic correlations between milking time and milk yield were 0.23, and the phenotypic ones were 0.27 [28]. Even higher values (0.36–0.47) of simple linear correlations were reported by Sitkowska et al. [23] and Sandrucci et al. [34]. Our study showed statistical correlations between milking speed and lactation milk yield (r = around 0.1 regardless of the period). The literature provides much stronger relationships between milking speed and yield; for example, Edwards et al. [28] reported genetic correlations of 0.39 and phenotypic correlations as high as 0.55.

In the next stage of study, for prediction of the cows’ milk yield based on periparturient data recorded by Lely milking robots, we used the decision tree method, which serves as a tool in making optimal decisions. According to Piwczyński et al. [35], the decision tree method, through analysis of the graphical model, makes it possible to identify factors affecting certain productive traits of animals. Breeders can use the graphical model to find a leaf node with the best value of a given trait and then, following the division path, reach the root node while identifying factors and their levels which affect that trait.

Table 5 identifies the variables used to construct the graphical model of the decision tree, which describes lactation milk yield expressed by their magnitude and number of divisions made on their basis. The obtained results indicate that lactation milk yield was mostly dependent on whether a cow survived to the next calving or was prematurely culled; the importance of this variable was 1 (on a scale of 0–1). Two divisions were made based on milking time (from 22 to 28 and from 5 to 7 days of lactation), and the other factors making up the tree were: number of milkings/day from 22 to 28 days of lactation, milking speed from 8 to 14 days of lactation and colostrum protein content, but the importance of these variables was lower and ranged from 0.2183 to 0.7783.

**Figure 2:**
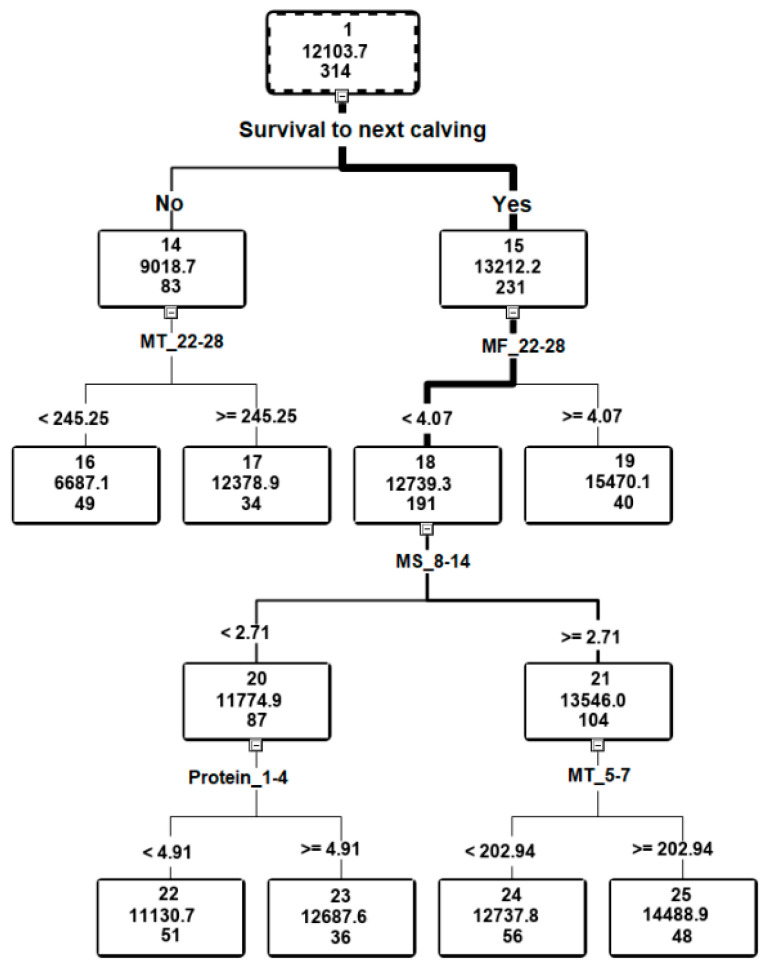
Diagram of decision tree. Abbreviations: MF_22–28—number of milkings/day during 22–28 days of lactation (no./day), MS_8–14—milking speed during 8–14 days of lactation (kg/min), MT_5–7—milk time during 5–7 days of lactation (s/day), MT_22–28—milk time during 22–28 days of lactation (s/day), Protein_1–4—protein content during 1–4 days of lactation (%).

The decision tree model for lactation yield of a cow contained 4 levels and 6 leaves (Figure 2). The information presented in the Figure 2 refers to the training set. The algorithm responsible for decisions of the tree showed that the most important variable differentiating the set was survival to next calving (Node 14, 15). Culling of the cows before the next calving (Node 14) naturally resulted in their lower milk yield. The node formed by the culled cows (26% of general) branched according to milking time during 22–28 days of lactation, and for this variable the threshold value was 245.25 s/day. Higher milk yield was achieved by the cows with longer milking times, and their advantage over cows with shorter milking times was 50%. The subset of cows that survived to the next calving (Node 15) was branched according to the number of milkings/day during 22–28 days of lactation into <4.07 (Node 18) and ≥4.07 (Node 19 became a leaf node). Higher lactation yield was achieved by the cows that were milked more frequently per day (15,470.1 kg vs. 12,739.3 kg). This supports the results of other authors [36]. Sorensen et al. [37] concluded that milking frequency is one of the determinants of the cow’s milk yield. More frequent milking increases their productivity and improves lactation persistency. Piwczyński et al. [35], who used decision tree technique to determine factors responsible for high monthly yield of AMS-milked cows, showed milking frequency to be the most important factor. According to Lyons et al. [38], increased milking frequency does result in higher milk yield of the cows, but this effect is largely dependent on the stage of lactation and udder health. Castro et al. [39] concluded that the optimal number of milkings/day is 2.4–2.6. In our study, the highest lactation yield was obtained by the cows which were milked ≥4.07 times/day during 22–28 days of lactation, whereas in the study by Piwczyński et al. [35], the highest daily yield was achieved by the cows milked ≥3.87 times/day. Hogeveen et al. [36] observed that the effect of milking frequency on milk yield was higher for higher yielding cows compared to lower producing cows.

The factor differentiating lactation yield of the cows with lower milking frequency (Node 18) was milking speed during 8–14 days of lactation (<2.71 kg/min (Node 20) and ≥2.71 kg/min (Node 21)); higher yield was achieved by the faster milkers and the milk yields were more equalized (11,774.9 kg vs. 13,546.0 kg). In herds in which milking parlours or milking robots are used, it is essential that cows are milked easily and quickly [40]. Tremblay et al. [41] stressed the importance of milking speed, because faster milkers occupy the AMS for a shorter time, thus contributing to its more efficient use. The results for division of Node 20 indicate that higher lactation milk yield was achieved by the cows whose colostrum contained more protein (≥4.91%). Probably leaf node 23 was formed by multiparous cows, whose colostrum was richer in protein, and in addition their yield was higher. In turn, the results of the last division of Node 21 indicate that higher lactation milk yield was achieved by the cows whose milkings during 5–7 days of lactation were longer (≤202.93 s/day).

It follows from the decision tree model that the highest full lactation milk yield (15,470.1 kg) was obtained by the cows milked ≥4.07 times/day from 22 to 28 days of lactation. For the other cows that survived to the next calving, the highest yields (14,488.9 kg) were assigned to leaf node 25, which was formed by the following divisions: milking speed (≤2.71 kg/min), milking time during 5–7 days of lactation (≥202.94 s/day). Sitkowska et al. [42] also showed that increased number of milkings and longer milking times were associated with higher milk yield. In turn, according to Wethal and Heringstad [9], the desirable traits are short milking time and ability to quickly leave the AMS after the last teat cup is removed.

## 4. Conclusions

The increasing rumination time and milking time/visit, as well as the increasing intake of AMS concentrate contributed to an increase in lactational milk yield, and the magnitude of these relationships increased with each week of lactation. Useful for predicting the milk yield per lactation was also the number of milkings/day.

The decision tree method showed that the most important factors responsible for lactation yield of the AMS cows was, in descending order of importance: survival to the next calving, milking time/visit and number of milkings/day (22–28 days of lactation), milking speed (8–14 days of lactation), milking time/visit (5–7 days of lactation) and protein content of colostrum. We proved that the application of the decision tree method could allow breeders to select, already in the postparturient period, appropriate levels of AMS milking variables, which will ensure high milk yield per lactation.

## Figures and Tables

**Table 1:** General characteristics of the studied herds.

Herd	Number of Milking Robots	Number of Cows in Barn	Technological Groups	Number of Cows/Robot	Feeding System	Housing System
A	1	65	In lactation, dry period	52–55	Partial Mixed Ration	free stalls, grates
B	3	225	In lactation, dry period	65–68	Partial Mixed Ration	free stalls, grates
C	2	150	In lactation, dry period	64–67	Partial Mixed Ration	free stalls, grates

**Table 2:** Descriptive statistics of rumination, milking parameters and colostrum traits in the period from 1 to 4 days of lactation.

Trait	N	$\bar{x}$	SD	CV (%)
Rumination time (min/day)	2665	326.76	125.77	38.49
Quantity of programmed concentrate feed (kg/day)	2675	3.26	0.65	19.86
Amount of concentrate intake (kg/day)	2669	2.56	1.03	40.16
Number of milkings/day	2603	1.82	0.60	33.21
Number of refusal milkings/day	2603	1.15	2.89	251.79
Automatic milking system (AMS) box time (s/visit)	2626	419.37	138.19	32.95
Milking time (s/visit)	2626	322.04	135.24	42.00
Time of colostrum flow from the udder quarter (s/visit)	2626	222.24	99.27	44.67
Dead milking time for udder quarters (s/visit)	2626	13.48	6.28	46.61
Colostrum milking speed (kg/min)	2613	2.78	1.14	40.95
Eectrical conductivity of colostrum (μS/cm)	2613	69.35	5.45	7.86
Colostrum temperature (°C)	2613	38.96	1.09	2.79
Colostrum yield (kg/day)	2591	17.91	9.62	53.75
Fat content (%)	2170	4.89	1.60	32.77
Protein content (%)	2170	4.85	0.46	9.59
Fat/protein ratio	2170	1.02	0.33	32.52

$\bar{x}$—mean, SD—standard deviation, CV—coefficient of variation.

**Table 3:** Descriptive statistics of rumination, milking parameters and milk traits in the period from 5 to 28 days of lactation.

Trait	N	$\bar{x}$	SD	CV (%)
Rumination time (min/day)	15,390	444.90	81.00	18.4
Quantity of programmed concentrate feed (kg/day)	15,466	6.19	1.68	27.1
Amount of concentrate intake (kg/day)	15,408	5.56	1.77	31.9
Number of milkings/day	15,285	2.78	0.85	30.52
Number of refusal milkings/day	15,336	1.70	3.21	188.4
AMS box time (s/visit)	15,285	446.43	143.80	32.2
Milking time (s/visit)	15,285	351.97	144.33	41.0
Time of milk flow from the udder quarter (s/visit)	15,285	250.41	106.14	42.4
Dead milking time for udder quarters (s/visit)	15,285	14.13	6.81	48.2
Milking speed (kg/min)	15,267	2.81	1.07	38.2
Electrical conductivity of milk (μS/cm)	15267	68.77	4.93	7.2
Milk temperature °C	15,267	39.03	0.75	1.92
Milk yield (kg/day)	15,359	35.23	11.51	32.7
Fat content (%)	13,295	4.00	0.76	18.9
Protein content (%)	13,295	3.54	0.33	9.4
Fat/protein ratio	13,295	1.14	0.23	20.1

$\bar{x}$—mean, SD—standard deviation, CV—coefficient of variation.

**Table 4:** Pearson correlation coefficients between rumination time, milking parameter and milk traits in in different periparturient periods and lactational milk yield.

Days of Periparturient Period	Rumination Time (min/day)	Electrical Conductivity of Colostrum/Milk (μS/cm)	Colostrum /Milk Temperature (°C)	Amount of Concentrate Intake (kg/day)	Number of Milkings /Day	Milking Time (s/visit)	Colostrum /Milking Speed (kg/min)
–14 to –8	0.126 ^xx^						
–7 to –1	0.111 ^x^						
1 to 4	0.097 ^x^	−0.060	0.002	0.109 ^x^	0.257 ^xx^	0.116 ^xx^	0.110 ^x^
5 to 7	0.249 ^xx^	−0.078	−0.003	0.101 ^x^	0.279 ^xx^	0.135 ^xx^	0.089 ^x^
8 to 14	0.312 ^xx^	−0.083	−0.021	0.136 ^xx^	0.301 ^xx^	0.155 ^xx^	0.082
15 to 21	0.307 ^xx^	−0.081	−0.010	0.150 ^xx^	0.280 ^xx^	0.168 ^xx^	0.100 ^x^
22 to 28	0.310 ^xx^	−0.086 ^x^	−0.015	0.197 ^xx^	0.252 ^xx^	0.174 ^xx^	0.107 ^x^

^xx^*p* < 0.01, ^x^ *p* < 0.05.

**Table 5:** The importance of tested variables based on “Importance” measure.

Variable	Number of Devisions	Importance
Survival to next calving	1	1
Milking time during 22–28 days of lactation (s/visit)	1	0.7782
Number of milkings/day during 22–28 days of lactation (no./day)	1	0.4793
Milking speed during 8–14 days of lactation (kg/min)	1	0.3720
Milking time during 5–7 days of lactation (s/visit)	1	0.2717
Protein content during 1–4 days of lactation (%)	1	0.2183

## Data Availability

3rd Party Data.

## References

[1] Barkema H.W., von Keyserlingk M.A.G., Kastelic J.P., Lam T.J.G.M., Luby C., Roy J.-P., LeBlanc S.J., Keefe G.P., Kelton D.F. (2015). Invited review: Changes in the dairy industry affecting dairy cattle health and welfare. J. Dairy Sci..

[2] Egger-Danner C., Cole J.B., Pryce J.E., Gengler N., Heringstad B., Bradley A., Stock K.F. (2014). Invited review: Overview of new traits and phenotyping strategies in dairy cattle with a focus on functional traits. Animal.

[3] Carlström C., Strandberg E., Johansson K., Pettersson G., Stålhammar H., Philipsson J. (2014). Genetic evaluation of in-line recorded milkability from milking parlors and automatic milking systems. J. Dairy Sci..

[4] Antanaitis R., Žilaitis V., Juozaitiene V., Noreika A., Rutkauskas A. (2018). Evaluation of rumination time, subsequent yield, and milk trait changes dependent on the period of lactation and reproductive status of dairy cows. Pol. J. Vet. Sci..

[5] Soriani N., Panella G., Calamari L. (2013). Rumination time during the summer season and its relationships with metabolic conditions and milk production. J. Dairy Sci..

[6] Stone A.E., Jones B.W., Becker C.A., Bewley J.M. (2017). Influence of breed, milk yield, and temperature-humidity index on dairy cow lying time, neck activity, reticulorumen temperature, and rumination behavior. J. Dairy Sci..

[7] Calamari L., Soriani N., Panella G., Petrera F., Minuti A., Trevisi E. (2014). Rumination time around calving: An early signal to detect cows at greater risk of disease. J. Dairy Sci..

[8] Liboreiro D.N., Machado K.S., Silva P.R.B., Maturana M.M., Nishimura T.K., Brandão A.P., Endres M.I., Chebel R.C. (2015). Characterization of peripartum rumination and activity of cows diagnosed with metabolic and uterine diseases. J. Dairy Sci..

[9] Wethal K.B., Heringstad B. (2019). Genetic analyses of novel temperament and milkability traits in Norwegian Red cattle based on data from automatic milking systems. J. Dairy Sci..

[10] Sitkowska B., Piwczyński D., Aerts J., Kolenda M., Özkaya S. (2017). Detection of high levels of somatic cells in milk on farms equipped with an automatic milking system by decision trees technique. Turkish J. Vet. Anim. Sci..

[11] Piwczyński D., Sitkowska B., Kolenda M., Brzozowski M., Aerts J., Schork P.M. (2020). Forecasting the milk yield of cows on farms equipped with automatic milking system with the use of decision trees. Anim. Sci. J..

[12] Piwczyński D., Nogalski Z., Sitkowska B. (2013). Statistical modeling of calving ease and stillbirths in dairy cattle using the classification tree technique. Livest. Sci..

[13] Siatka K., Sawa A., Piwczyński D., Bogucki M., Krężel-Czopek S. (2018). Factors affecting first insemination success in Polish Holstein-Fresian cows. Anim. Sci. Pap. Reports.

[14] Ghiasi H., Piwczyński D., Khaldari M., Kolenda M. (2016). Application of classification trees in determining the impact of phenotypic factors on conception to first service in Holstein cattle. Anim. Prod. Sci..

[15] Bisinotto R.S., Greco L.F., Ribeiro E.S., Martinez N., Lima F.S., Staples C.R., Thatcher W.W., Santos J.E.P. (2012). Influences of nutrition and metabolism on fertility of dairy cows. Anim. Reprod..

[16] SAS Institute Inc. (2014). SAS Institute Inc.: SAS/STAT® 9.4 User’s Guide Cary.

[17] Schirmann K., Chapinal N., Weary D.M., Vickers L., Von Keyserlingk M.A.G. (2013). Short communication: Rumination and feeding behavior before and after calving in dairy cows. J. Dairy Sci..

[18] Schirmann K., Chapinal N., Weary D.M., Heuwieser W., von Keyserlingk M.A.G. (2011). Short-term effects of regrouping on behavior of prepartum dairy cows. J. Dairy Sci..

[19] Hovinen M., Pyörälä S. (2011). Invited review: Udder health of dairy cows in automatic milking. J. Dairy Sci..

[20] Ontsouka C.E., Bruckmaier R.M., Blum J.W. (2003). Fractionized milk composition during removal of colostrum and mature milk. J. Dairy Sci..

[21] King M.T.M., LeBlanc S.J., Pajor E.A., Wright T.C., DeVries T.J. (2018). Behavior and productivity of cows milked in automated systems before diagnosis of health disorders in early lactation. J. Dairy Sci..

[22] Kaufman E.I., Asselstine V.H., LeBlanc S.J., Duffield T.F., DeVries T.J. (2018). Association of rumination time and health status with milk yield and composition in early-lactation dairy cows. J. Dairy Sci..

[23] Sitkowska B., Piwczyński D., Brzozowski M., Aerts J. (2016). Quarter milking in primiparous and multiparous cows*. Sci. Ann. Polish Soc. Anim. Prod..

[24] Unal H., Kuraloglu H., Koyuncu M., Alibas K. (2017). Effect of cow traffic type on automatic milking system performance in dairy farms. J. Anim. Plant Sci..

[25] Bogucki M., Sawa A., Kuropatwińska I. (2017). Association of automatic milking systems milking frequency in primiparous and multiparous cows with their yield and milkability. Acta Agric. Scand. A Anim. Sci..

[26] Piwczyński D., Gondek J., Sitkowska B., Kolenda M. (2020). Comparison of results coming from automatic milking system in selected countries in Europe and U.S. J. Cent. Eur. Agric..

[27] Piwczyski D., Sitkowska B., Aerts J., Kolenda M. (2013). The daily distribution of milkings of cows in farms equipped with the automatic milking system. Acta Sci. Pol. Zootech..

[28] Edwards J.P., Jago J.G., Lopez-Villalobos N. (2014). Analysis of milking characteristics in New Zealand dairy cows. J. Dairy Sci..

[29] Gáde S., Stamer E., Bennewitz J., Junge W., Kalm E. (2007). Genetic parameters for serial, automatically recorded milkability and its relationship to udder health in dairy cattle. Animal.

[30] Carlström C., Pettersson G., Johansson K., Strandberg E., Stålhammar H., Philipsson J. (2013). Feasibility of using automatic milking system data from commercial herds for genetic analysis of milkability. J. Dairy Sci..

[31] Guliński P., Kłopotowska A. (2019). An attempt to develop a method for determining the typical chemical composition of the milk of Polish Holstein-Friesian cows–a proposal. Rocz. Nauk. Pol. Tow. Zootech..

[32] Boas D.F.V., Filho A.E.V., Pereira M.A., Junior L.C.R., Faro L. (2017). El Association between electrical conductivity and milk production traits in dairy Gyr cows. J. Appl. Anim. Res..

[33] Neamț R., Ilie D.E., Gavojdian D., Acatincăi S., Florin N., Cziszter L. (2016). Influence of Electrical Conductivity, Days in Milk and Parity on Milk Production and Chemical Composition. Anim. Sci. Biotechnol..

[34] Sandrucci A., Tamburini A., Bava L., Zucali M. (2007). Factors Affecting Milk Flow Traits in Dairy Cows: Results of a Field Study. J. Dairy Sci..

[35] Piwczyński D., Brzozowski M., Sitkowska B. (2020). The impact of the installation of an automatic milking system on female fertility traits in Holstein-Friesian cows. Livest. Sci..

[36] Hogeveen H., Ouweltjes W., De Koning C.J.A.M., Stelwagen K. (2001). Milking interval, milk production and milk flow-rate in an automatic milking system. Livest. Prod. Sci..

[37] Sorensen A., Muir D.D., Knight C.H. (2008). Extended lactation in dairy cows: Effects of milking frequency, calving season and nutrition on lactation persistency and milk quality. J. Dairy Res..

[38] Lyons N.A., Kerrisk K.L., Garcia S.C. (2014). Milking frequency management in pasture-based automatic milking systems: A review. Livest. Sci..

[39] Castro A., Pereira J.M., Amiama C., Bueno J. (2012). Estimating efficiency in automatic milking systems. J. Dairy Sci..

[40] Szymik B., Topolski P., Jagusiak W. (2018). Cechy zdolności udojowej–cechy funkcjonalne istotne w nowoczesnych systemach doju. Wiadomości Zootech..

[41] Tremblay M., Hess J.P., Christenson B.M., McIntyre K.K., Smink B., van der Kamp A.J., de Jong L.G., Döpfer D. (2016). Factors associated with increased milk production for automatic milking systems. J. Dairy Sci..

[42] Sitkowska B., Piwczynski D., Aerts J., Waskowicz M. (2015). Changes in milking parameters with robotic milking. Arch. Tierzucht.

